# Pigmented Bowen’s Disease: A Case Report in Saudi Arabia

**DOI:** 10.7759/cureus.66191

**Published:** 2024-08-05

**Authors:** Afnan Hasanain, Abdulmonem Almutawa, Fahad Aljuaid, Ahmad M Tayeb, Jumanah A Bahattab, Luai Assaedi, Khalid Alshareef

**Affiliations:** 1 Department of Dermatology, King Faisal Specialist Hospital & Research Centre, Jeddah, SAU; 2 Department of Pathology and Laboratory Medicine, King Faisal Specialist Hospital & Research Centre, Jeddah, SAU; 3 Dermatology Training Joint Program in Western Region, Ministry of Health, Jeddah, SAU; 4 College of Medicine, King Saud bin Abdulaziz University for Health Sciences, Jeddah, SAU

**Keywords:** histopathology, dermatology case report, squamous cell carcinoma in situ, pigmented bowen's disease, dermatoscopy

## Abstract

Bowen’s disease (BD) is an in situ squamous cell carcinoma of the epidermis with multiple etiologies and a high incidence among Caucasians. It commonly occurs in photo-exposed areas of the skin, although other sites can also be affected. Most lesions are solitary, and their morphology can vary based on the lesion’s age, origin, and degree of keratinization. A 50-year-old female from Saudi Arabia presented to the dermatology clinic with a three-year history of slowly enlarging skin lesions on the left side of her chest. Initially, the lesion appeared three years ago, but she observed changes and a darkening in color over the past year, accompanied by mild pain and itching. On examination, the lesion was a 2 × 2 cm, well-defined, unevenly pigmented brown-black plaque with a dispersed pigment pattern and irregular borders with globularity on the left side of the upper chest. A 4 mm punch biopsy was taken from the most pigmented area and sent for histopathological examination, which confirmed the diagnosis of pigmented BD.

## Introduction

Bowen’s disease (BD), also known as squamous cell carcinoma (SCC) in situ, primarily affects the elderly. It typically presents as a well-demarcated erythematous plaque with a crusty and/or scaly surface. Risk factors include sunlight exposure, fair skin, immunosuppression, chronic arsenic exposure or ingestion, human papillomavirus, and genetic susceptibility [1]. Occasionally, when Bowen’s lesion exhibits hyperpigmentation, it is referred to as pigmented BD (PBD), a rare subtype. One study reviewing 420 BD lesions found seven cases of PBD, accounting for 1.67% of the total lesions [2]. Differential diagnoses for a PBD lesion include malignant melanomas, seborrheic keratoses, actinic keratoses, bowenoid papulosis, and basal cell carcinomas. Accurate diagnosis requires histopathology, dermoscopy, and correlation with clinical history and examination [1]. We report a case of a 50-year-old female with a pigmented skin lesion on the left breast. The initial working diagnosis was melanoma versus atypical nevus, but the biopsy revealed it to be PBD.

## Case presentation

**Figure 1:**
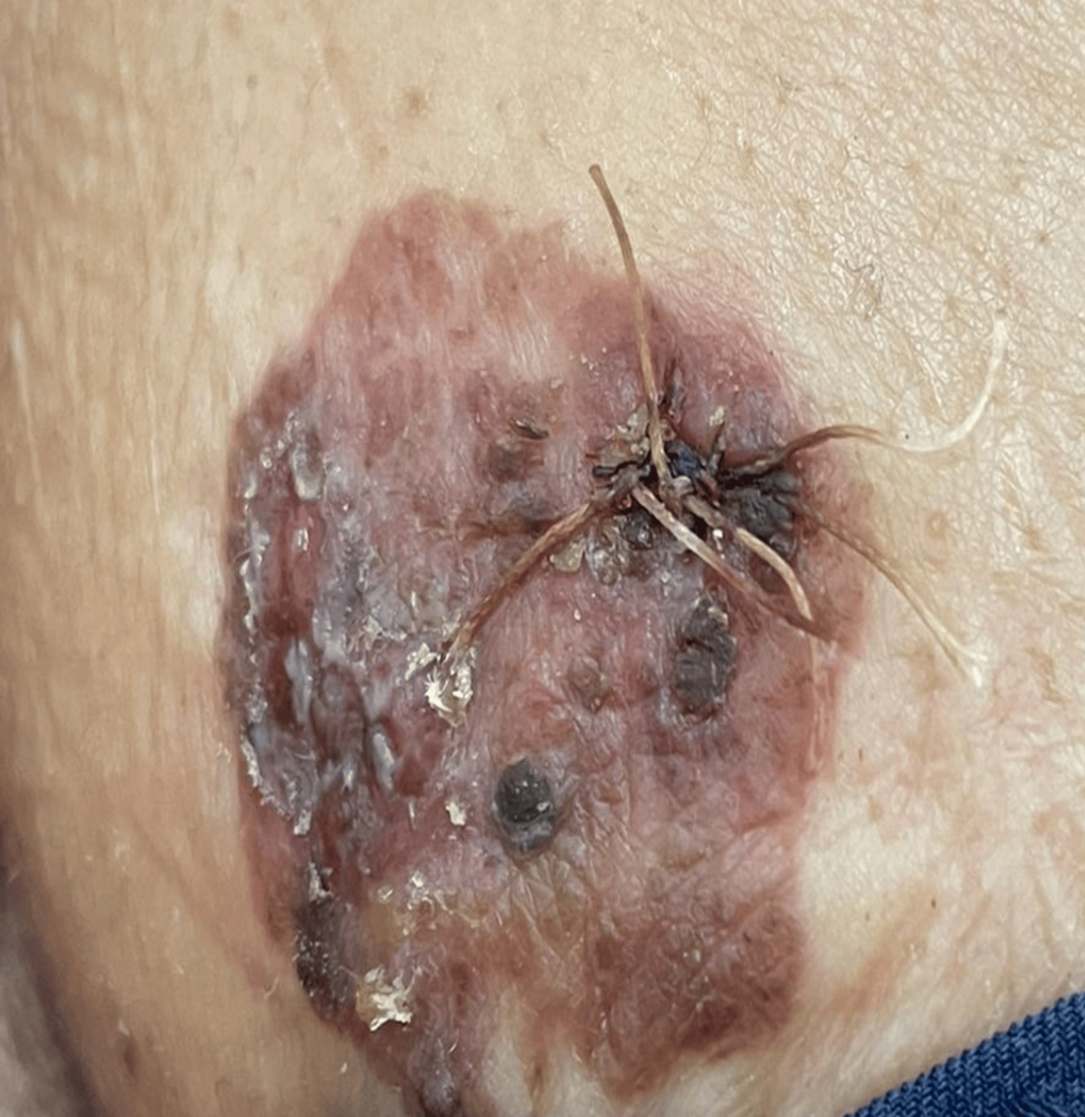
A 2 × 2 cm well-defined, unevenly pigmented brown-black plaque with a dispersed pigment pattern, irregular borders, and globularity, located on the left side of the upper chest A 3-0 Vicryl suture was placed following the punch biopsy. Erosion on the left side of the lesion was noted only during the second visit.

Our patient was a 50-year-old female from Saudi Arabia with skin type IV and no significant medical history. She was referred to our dermatology clinic with a three-year history of a slowly enlarging skin lesion on the left side of her chest. The lesion had started three years prior, but she noticed that it had been changing and darkening in color over the past year. Additionally, it had become associated with mild pain and itching. There was no family or personal history of skin cancers, and there was no history of prolonged sun exposure. On examination, a 2 × 2 cm well-defined, unevenly pigmented brown-black plaque with a dispersed pigment pattern, irregular borders, and globularity was observed on the left side of the upper chest (Figure 1).

**Figure 2:**
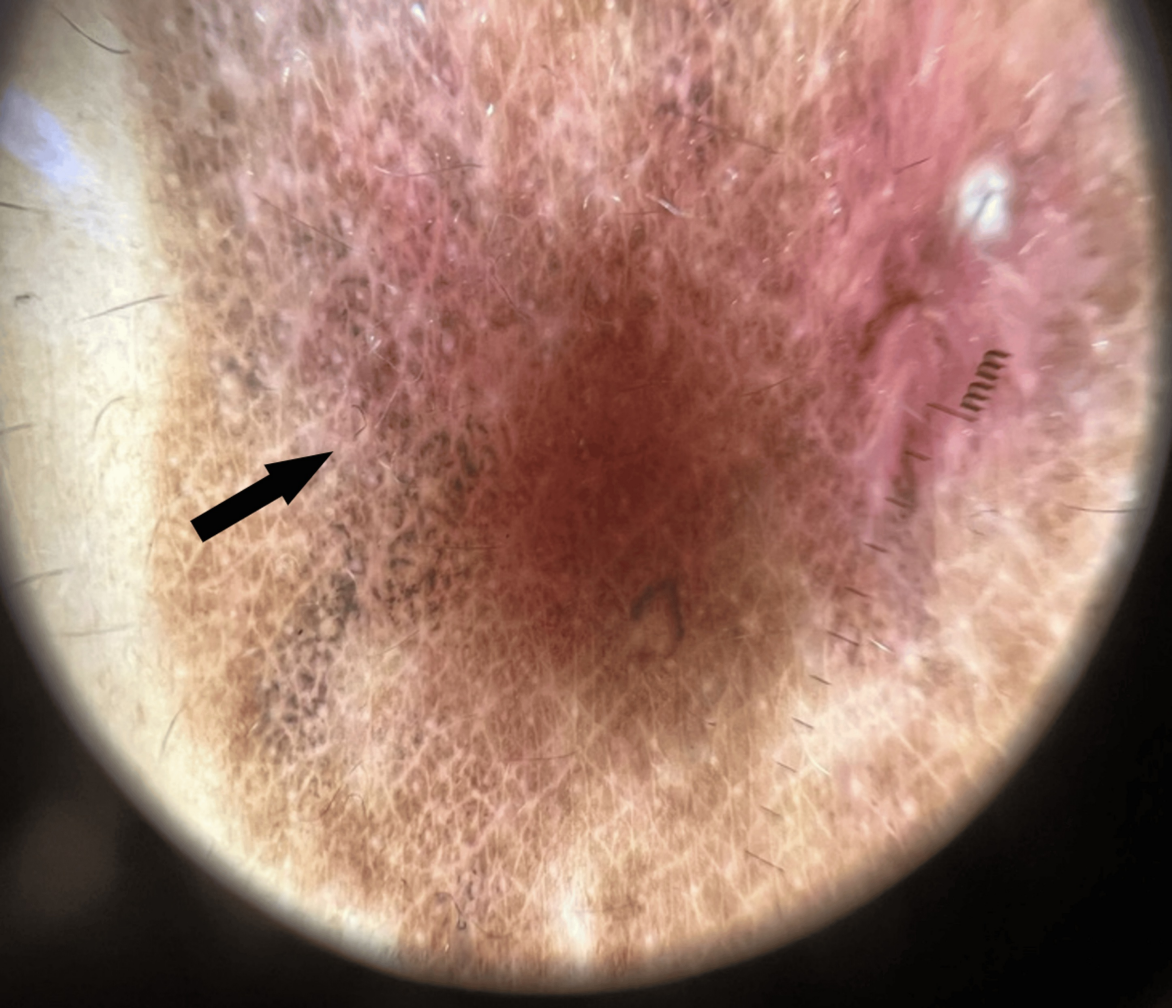
Light and dark globules with ill-defined erythema (arrow)

No other skin lesions were observed. The nails and mucous membranes were all normal. Dermoscopic examination showed light and dark brown globules with ill-defined erythema (Figure 2).

**Figure 3:**
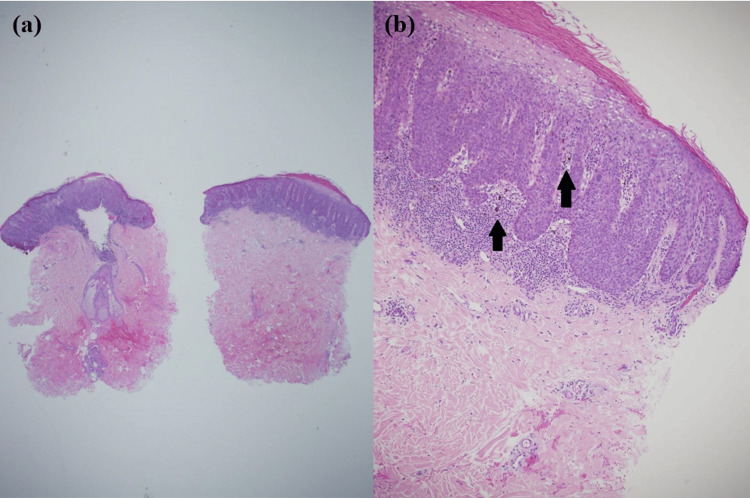
Histological features of the sample (a) Scanning magnification photomicrograph showing an acanthotic and dark-appearing epidermis with elongated rete ridges. A band of dense lymphocytic inflammatory response is observed beneath it (H&E stain; original magnification: 20×). (b) A full-thickness lack of epidermal keratinocyte maturation is noted. A parakeratotic layer is present on top, with melanin pigments deposited in the papillary dermis (arrows) (original magnification: 100×).

A 4 mm punch skin biopsy was taken from the most pigmented part and sent for histopathological examination. The scanning magnification photomicrograph showed an acanthotic and dark-appearing epidermis with elongated rete ridges. A band of dense lymphocytic inflammatory responses was observed beneath it (Figure 3A). A full-thickness lack of epidermal keratinocyte maturation was noted. A parakeratotic layer was present on top, and some melanin pigments were deposited in the papillary dermis (arrows) (Figure 3B).

**Figure 4:**
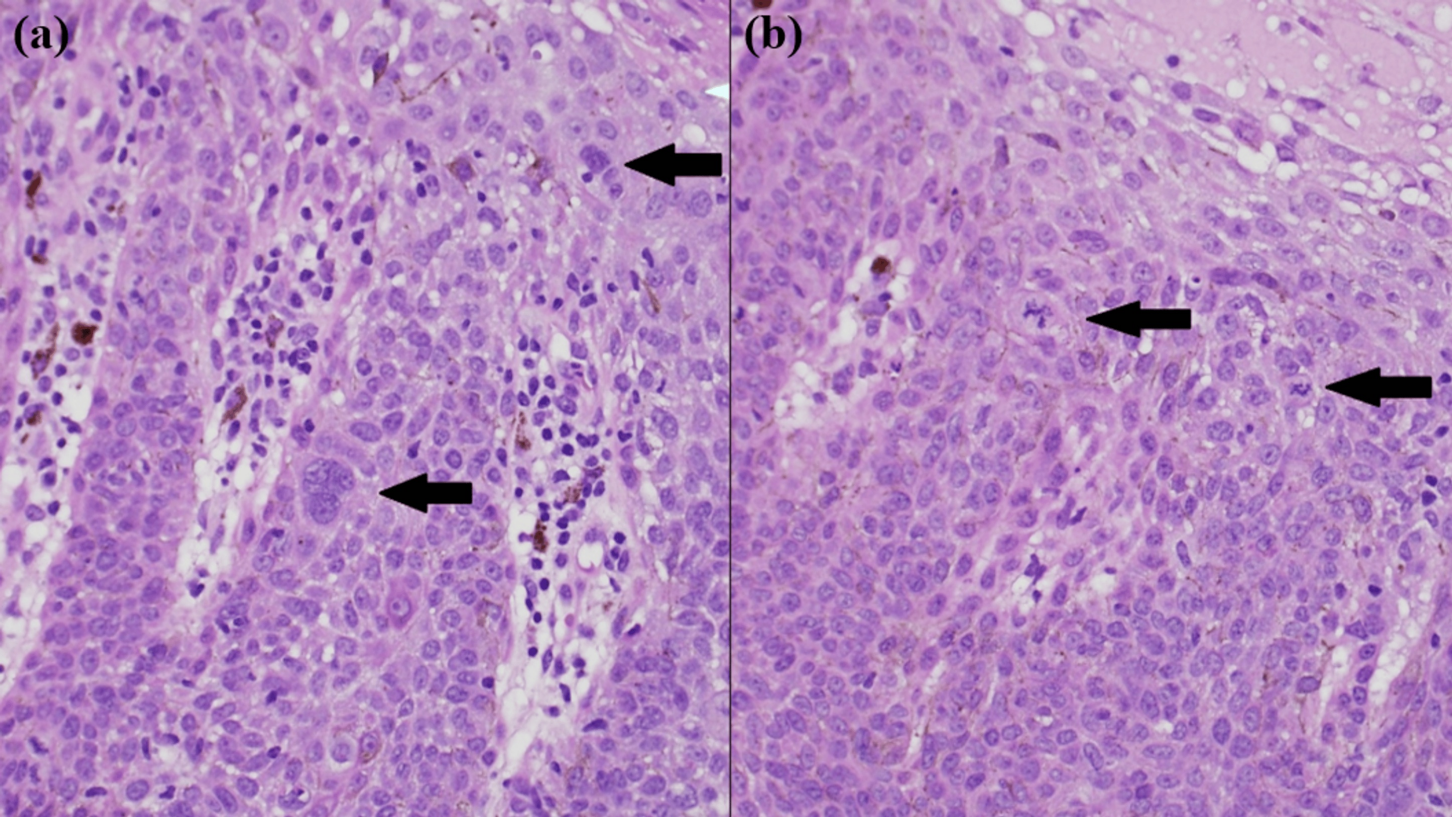
High-power histological features (a) High-power photomicrograph illustrating highly atypical and multinucleated keratinocytes (arrows) (H&E stain; original magnification: 400×). (b) Two atypical mitotic figures located at a high level within the epidermis (arrows) (H&E stain; original magnification: 400×).

High-power photomicrographs illustrated (Figure 4A) some keratinocytes that were highly atypical and multinucleated (arrows). Subepidermal melanin pigment deposition was noted, along with melanin within some of the upper epidermal atypical keratinocytes. In Figure 4B, two atypical mitotic figures were located at a high level within the epidermis (arrows).

No laboratory tests were performed. A diagnosis of PBD (SCC in situ, SCCIS) was confirmed. The patient was referred to plastic surgery for a complete excision. Imiquimod was prescribed to be applied three times per week for four weeks. After the excision, the sample was sent to the histopathologist, who reported no residual SCC. Changes in the dermal scar, inflammation, and melanin pigment incontinence were noted. Scattered atypical lymphocytes with mild cellular atypia were observed in the epidermis. These lymphocytes were likely reactive and probably appeared as a result of imiquimod treatment. Follow-up in the clinic was scheduled for three months, and then every three months for three years.

## Discussion

BD is a subtype of intraepidermal SCC that develops in sun-exposed parts of the skin and occurs at variable incidence across populations and ethnic groups, with a relatively higher prevalence in white Caucasian individuals and females [3-5]. By contrast, PBD, a rare subtype of SCCIS, is described as more prevalent in men and individuals with darker skin [6]. The hyperpigmentation of PBD is due to several factors, notably the higher melanocyte hyperplasia compared with the other types and the presence of well-differentiated atypical keratinocytes, which produce melanocyte-stimulating cytokines [7]. In the case of our patient, keratinocytes were highly atypical with subepidermal and epidermal melanin deposition.

Dermoscopy examination represents a useful tool in intraepidermal carcinoma, including PBD. Characteristic clustered vessels have been described in the center of the lesion, mimicking the shape of the renal glomerular apparatus. Additionally, brown, linearly arranged dots are often observed in the periphery [8]. In a series of 21 cases of BD, including 11 non-pigmented and 10 pigmented, dermoscopy revealed “glomerular” vessels in all non-pigmented cases and eight out of the 10 pigmented ones. The other characteristic feature was the scaly surface. Furthermore, PBD showed another feature, including “small brown globules, regularly packed in a patchy distribution” [9]. Dermoscopy findings in our case showed light and dark brown globules with ill-defined erythema.

The diagnosis of the present case of PBD was based on the biopsy findings, showing typical aspects of acanthosis, hyperkeratosis, and keratinocyte atypia in the epidermis that respect the dermoepidermal junction [7]. A punch biopsy was performed, which is preferred over a curette biopsy. Indeed, a punch biopsy enables viewing the full epidermis and dermis thickness, thereby establishing whether there is any invasive disease. However, biopsy remains limited by the probability of missing the tumor spots where invasiveness may be underlying. A five-year retrospective study that reviewed Mohs micrographic surgery slides of 566 operated cases of biopsy-proven SCCIS revealed that 92 (16.3%) of these cases were invasive SCC [10]. Another study involving 29 consecutive patients treated for SCCIS found that nine (31%) had invasive SCC on postoperative histology [11]. This highlights the importance of performing a postoperative histological examination to confirm the clearance of the lesion.

Interestingly, certain clinical and histological factors were demonstrated to predict the invasiveness of biopsy-proven SCCIS, among them a preoperative diameter of 14 mm [11]. This was the case of our patient, whose lesion was measured at 20 × 20 mm preoperatively, which indicates the need for closer follow-up with the patient.

While SCCIS constitutes a less malignant type, due to its noninvasiveness and slow growth, it is considered a precursor for invasive SCC with a risk of 3-5% [5,12]. This emphasizes the interest in early diagnosis to prevent such a progression. The present case of PBD in a female patient with skin type IV was diagnosed after three years of onset. The morphological changes and the occurrence of itchiness were probably the principal triggers for the specialist consultation. Furthermore, SCCIS is associated with a 4- to 8-fold incidence ratio for other nonmelanoma skin cancers that have sunlight exposure as a common risk factor [13]. Therefore, patients diagnosed with SCCIS should benefit from meticulous skin examination and careful follow-up. In the case of our patient, we have scheduled a trimestral follow-up in the dermatology clinic for three years.

The treatment of SCCIS comprises several options. Nevertheless, the management approach should consider both the patient’s and the tumor’s characteristics. Patient characteristics that may impact the treatment choice or outcome include immune status, baseline medical history, adherence to treatment, and esthetic issues. Tumor characteristics include size, site, and the number of lesions. Conservative methods use topical chemotherapy, such as imiquimod and fluorouracil, and photodynamic, laser, or radiation therapy. Invasive methods include surgical excision, Mohs micrographic surgery, and curettage [7]. In our patient, we used a combination of surgical excision and imiquimod. After treatment, patients should also be followed up for any recurrence. A population-based study from Hawaii showed that BD is associated with a 1.4% risk of recurrence after treatment [4].

## Conclusions

PBD is a rare subtype of SCCIS characterized by hyperpigmentation and may be underdiagnosed compared to other subtypes. There is a need to raise physicians’ awareness about BD and its different clinical presentations to enhance early diagnosis and reduce the risk of progression to invasive SCC.
